# Differences in Metabolite Profiles and Bioactivities of Intra-Strain Variants of Marine Fungus *Penicillium antarcticum* KMM 4668

**DOI:** 10.3390/metabo15020077

**Published:** 2025-01-27

**Authors:** Gleb V. Borkunov, Natalya N. Kirichuk, Viktoria E. Chausova, Roman S. Popov, Olesya I. Zhuravleva, Ekaterina A. Chingizova, Ekaterina A. Yurchenko, Marina P. Isaeva, Anton N. Yurchenko

**Affiliations:** 1 G.B. Elyakov Pacific Institute of Bioorganic Chemistry, Far Eastern Branch of the Russian Academy of Sciences, 159 Prospect 100-Letiya Vladivostoka, Vladivostok 690022, Russiapopov_rs@piboc.dvo.ru (R.S.P.); chingizova_ea@piboc.dvo.ru (E.A.C.); eyurch@piboc.dvo.ru (E.A.Y.); issaeva@piboc.dvo.ru (M.P.I.); 2 Laboratory of Biologically Active Compounds, Institute of High Technologies and Advanced Materials, Far Eastern Federal University, Vladivostok 690922, Russia

**Keywords:** *Penicillium*, intra-strain phenotypic diversity, multi-locus phylogenetic analysis, UHPLC-MS, cytotoxic activity

## Abstract

**Background:** During the cultivation of the marine fungus KMM 4668 on solid agar medium, the morphological instability of the strain was observed. As a result of the selection work, five intra-strain variants, named KMM 4711, KMM 4712, KMM 4713, KMM 4714, and KMM 4715, were obtained. **Methods:** The main objectives of this work were to compare the parent strain and its intra-strain variants using multi-locus phylogenetic analysis and to study the UPLC MS metabolite profiles and cytotoxic activities of their extracts. **Results:** A study of the original strain, KMM 4668, and its intra-strain variants using multi-locus phylogenetic analysis showed that they are sequence identical and belong to *Penicillium antarcticum*. UPLC MS metabolite profiling of fungal extracts revealed 20 compounds, including cladosporin-related polyketides, carotane sesquiterpenoids, andrastine meroterpenoids, and alkaloids. It was shown that the intra-strain variants KMM 4713 and KMM 4714 differed most strongly from the others in the increased production of cladosporin-related compounds, carotanoids, and the alkaloid chrysogin. In addition, the influence of fungal extracts on the viability of four mammalian cell lines was investigated. **Conclusions:** It has been shown that the intra-strain variants of *P. antarcticum* KMM 4668 may be promising sources of bioactive secondary metabolites.

## 1. Introduction

Marine-derived fungi are rich sources of promising molecules with diverse bioactive properties [1,2]. Most of them belong to 590 genera of Ascomycota, including globally occurring *Penicillium*, *Aspergillus*, and *Cladosporium*. *Penicillium* species are among the most widespread fungal organisms worldwide. *Penicillium antarcticum* is a common micromycete species belonging to the subgenus *Aspergilloides*, section *Canescentia*, and *Atroveneta* series [3]. Currently, the section *Canescentia* includes 23 species, 6 of which belong to the series *Atroveneta*, including *P*. *antarcticum* A.D. Hocking & C.F. McRae [4]. Along with other members of the section *Canescentia*, *P*. *antarcticum* is a characteristic species of terrestrial and marine fungal communities and is frequently isolated from soils and substrates of plant and animal origin [5]. According to the Global Biodiversity Information Facility (https://www.gbif.org, accessed on 24 July 2025), strains belonging to the *P. antarcticum* species were isolated from samples collected worldwide, but are mainly distributed in temperate and polar latitudes [4,6,7].

Various compounds have been reported from fungi of the section *Canescentia*, including the following polyketides: cladosporin (asperentin) and its various derivatives [8,9], penicanesins [10], atrovenetin [11], antarones [12]; diketopiperazines—*cis*-cyclo(4*R*-Hyp, L-Leu), *trans*-cyclo(4*R*-Hyp, L-Leu), and *cis*-cyclo(4*R*-Hyp, L-Phe) [13]; alkaloids—aurantioclavine [14] and chrysogine [15]; and andrastin-type meroterpenoids [16].

Previously, our research team isolated a range of *β*-resorcylic acid and cladosporin derivatives [17], as well as andrastin-type meroterpenoid meroantarctines A-C [18], from the fungus *P*. *antarcticum* KMM 4685, isolated from brown algae *Sargassum miyabei* (the Sea of Japan). Cyclopiane diterpenes, together with a new precursor of cladosporin, were obtained from the fungus *P. antarcticum* KMM 4670 (previously identified as *P. ochotense*) isolated from marine sediments [19], and carotane sesquiterpenoid piltunines A-F were isolated from the marine sediment-derived fungus *P. antarcticum* KMM 4668 (previously identified as *P. piltunense*) [20].

It has been reported that ten single-spore isolates were obtained from the aflatoxin-producing *Aspergillus parasiticus* strain M-3, and some of these isolates had a higher production of aflatoxin [21]. In another study, intra-strain variants of *Cryptococcus neoformans* with different colors were obtained using phloxin B medium [22]. At the same time, changes in the morphology of fungal strains of biotechnological importance, and the degeneration of bioactive compound production, are known [23], which is a matter of particular interest in relation to strains that produce important compounds.

Thus, the aim of the present work was to investigate the original *P. antarcticum* KMM 4668 and its intra-strain variants using multi-locus phylogenetic analysis and to study their UPLC MS metabolite profiles and the cytotoxic activities of their extracts against cancer and normal cells.

## 2. Materials and Methods

### 2.1. Fungal Intra-Strain Variant Selection

To obtain monoconidial cultures, a suspension of conidia of the original strain was prepared at a concentration of 4 × 10^2^ conidia/mL. Several drops of the suspension were evenly spread over the surface of wort agar in Petri dishes. The dishes were incubated at room temperature until colony growth was visible. Individual colonies were plated in a pure culture on slanted wort agar. The obtained cultures were sorted by colony appearance, and five of them, characterized by different phenotypic features, were selected for further investigation.

To study the phenotypic features, the obtained variants were plated on Petri dishes containing Czapek Yeast Autolysate agar medium (CYA) [24].

The fungal strains were stored in the Collection of Marine Microorganisms (PIBOC FEB RAS, Vladivostok, Russia) under the codes KMM 4668, KMM 4711, KMM 4712, KMM 4713, KMM 4714, and KMM 4715.

### 2.2. DNA Extraction, Amplification, and Sequencing

Genomic DNAs of strain KMM 4668 and its variants, KMM 4711, KMM 4712, KMM 4713, KMM 4714, and KMM 4715, were isolated from fungal mycelium grown on malt extract agar (MEA) at 25 °C for 7 days using a MagJET Plant Genomic DNA Kit (Thermo Fisher Scientific, Waltham, MA, USA) following the manufacturer’s protocol. PCR was performed using GoTaq Flexi DNA Polymerase (Promega, Madison, WI, USA). Amplification and sequencing of the ITS region, of the partial *BenA*, *CaM*, and *RPB2* genes, were performed as described in [19]. Approximately 600 bp fragments of the ITS region, about 500 bp fragments of *BenA,* and about 550 bp fragments of the *CaM* genes, as well as about 900 bp fragments of the *RPB2* gene (only for KMM 4668), were amplified. The fragments were purified and sequenced with gene-specific primers using a SeqStudio™ genetic analyzer (Thermo Fisher Scientific, Waltham, MA, USA). The obtained gene sequences were deposited in GenBank under the accession numbers indicated in Table S1.

### 2.3. Multi-Locus Phylogenetic Analysis

The ITS region, the partial *BenA* and *CaM* gene sequences of the fungal strains KMM 4668 and KMM 4711–KMM 4715, and members of the genus *Penicillium* section *Canescentia*, series *Atroveneta*, according to [3,25], were aligned using MEGA X software version 11.0.9 [26] using the Clustal W algorithm. A search for ITS, *BenA,* and *CaM* sequences of ex-type strains was performed in the GenBank database using the BLASTn algorithm (http://www.ncbi.nlm.nih.gov/BLAST, accessed on 10 September 2024). Multiple alignment of ITS, *BenA,* and *CaM* sequences of these strains and ex-type strains of the genus *Penicillium* section *Canescentia*, series *Atroveneta*, and their phylogenetic analysis were carried out using MEGA X software version 11.0.9 [26]. The phylogenetic tree was built based on the aligned combined sequences of ITS, *BenA,* and *CaM* using the ML algorithm and the selected optimal evolutionary model: the Kimura 2-parameter model [27]. A bootstrap test based on 1000 replicates was used to statistically estimate branch support. The sequences of the strain *Talaromyces marneffei* CBS 388.87^T^ were used as an outgroup for the phylogenetic analysis (Table S1).

### 2.4. Cultivation of Fungi

Each fungal strain (KMM 4668, KMM 4711, KMM 4712, KMM 4713, KMM 4714, and KMM 4715) was cultured on a wort agar medium at 22 °C for three weeks in one test tube (20 mL), containing 10 mL of the medium (2 mL of wort, 8 mL of natural seawater, 0.18 g of agar).

### 2.5. Extraction and HPLC MS Analysis

#### 2.5.1. Extraction of Fungal Cultures

Each fungal culture with medium was extracted with EtOAc (100 mL) and then evaporated in vacuo to yield a crude extract (Table S2). Each extract was then dissolved in methanol and passed through a column with C_18_-SiO_2_ (YMC Gel ODS-A). The masses of purified extracts are presented in Table S2.

#### 2.5.2. HPLC MS Analysis of Fungal Extracts

HPLC MS analysis was performed using a Bruker Elute UHPLC chromatograph (Bruker Daltonics, Bremen, Germany) connected to a Bruker Impact II Q-TOF mass spectrometer (Bruker Daltonics, Bremen, Germany). An InfinityLab Poroshell 120 SB-C18 column (2.1 × 150 mm, 2.7 m, Agilent Technologies, Santa Clara, CA, USA) was used for chromatographic separation. Chromatographic separation and mass spectrometric detection were performed as previously described [28].

#### 2.5.3. UHPLC-Q-TOF Data Analysis

UHPLC-Q-TOF was performed according to the method described in the Supplementary Materials.

### 2.6. Principal Component Analysis (PCA)

PCA, hierarchical dendrogram, and visualization of the resulting graphs were performed with MZMine (version 2.53) [29].

### 2.7. Cytotoxic Activity of Extracts

The human cervical cancer HeLa, prostate cancer PC-3, and breast cancer MCF-7 cells were purchased from ATCC (Manassas, VA, USA). Rat cardiomyocyte H9c2 cells were kindly provided by Prof. Dr. Gunhild von Amsberg from the Martini-Klinik Prostate Cancer Center, University Hospital Hamburg-Eppendorf, Hamburg, Germany. The cells were cultured in DMEM with 10% fetal bovine serum (BioloT, St. Petersburg, Russia) and 1% penicillin/streptomycin (BioloT, St. Petersburg, Russia). For the experiments, HeLa, PC-3, and MCF-7 cells were seeded at concentrations of 5 × 10^3^ cell/well, and H9c2 cells were seeded at concentrations of 3 × 10^3^ cell/well. Then, the experiments started after 24 h.

The cells were treated with the extracts at a concentration of 10 μg/mL for 24 h, and cell viability was measured using an MTT (3-(4,5-dimethylthiazol-2-yl)-2,5-diphenyltetrazolium bromide) assay, which was performed according to the manufacturer’s instructions (Sigma-Aldrich, St.-Louis, MO, USA). Culture plates were incubated for 24 h, and the OD_570_ was measured using a Multiskan FS spectrophotometer (Thermo Scientific Inc., Beverly, MA, USA). The results were calculated as a percentage of the control.

## 3. Results

### 3.1. Morphological Study

**Figure 1:**
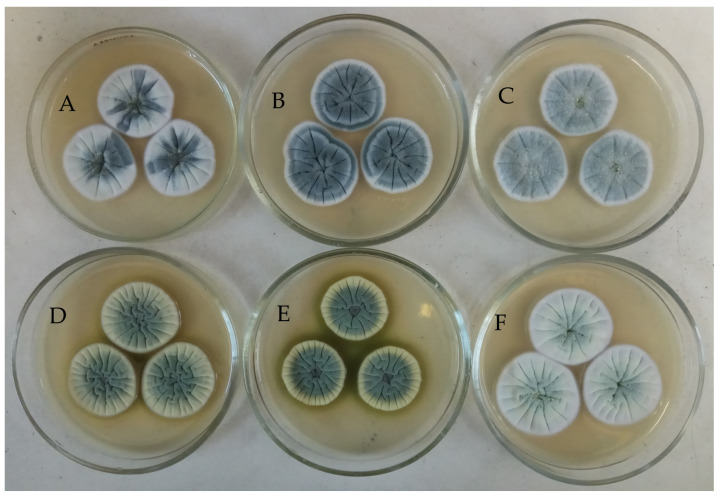
Morphology of intra-strain variants of *Penicillium antarcticum* KMM 4668 obtained by selection work ((**A**)—KMM 4668, (**B**)—KMM 4711, (**C**)—KMM 4712, (**D**)—KMM 4713, (**E**)—KMM 4714, (**F**)—KMM 4715).

During the study of the cultural and morphological characteristics of the KMM 4668 strain and its subsequent cultivation on solid agar medium in Petri dishes, the separation of recessive phenotypes in the form of sectors was observed. Because of the instability of the original KMM 4668 strain, selection work was carried out, and as a result, five intra-strain variants with various phenotypic features (color of sporulation and mycelium, sporulation level, presence of pigment) were isolated and named KMM 4711, KMM 4712, KMM 4713, KMM 4714, and KMM 4715 (Figure 1).

### 3.2. Molecular Identification of the Fungal Strain Variants

**Figure 2:**
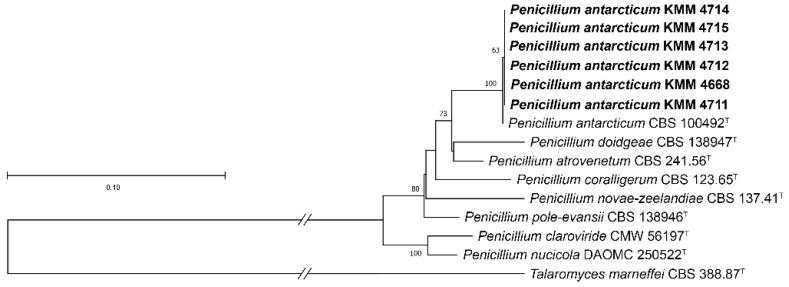
ML tree based on concatenated ITS-*BenA*-*CaM* gene sequences showing phylogenetic positions of the original strain KMM 4668, and its intra-strain variants KMM 4711-KMM 4715 among members of the genus *Penicillium* section *Canescentia*, series *Atroveneta*. Bootstrap values (%) of 1000 replications and nodes with confidence values greater than 50% are indicated. The scale bar represents 0.1 substitutions per site.

The original strain KMM 4668 and its intra-strain variants, KMM 4711 and KMM 4715, were identified using molecular markers such as ITS regions and partial *BenA* and *CaM* genes. Additionally, the *RPB2* gene was used to identify the original strain KMM 4668 (GenBank accession number: PQ336934). A BLAST search showed that the partial *BenA*, as well as the *RPB2* gene sequences of strain KMM 4668, were 100% identical to the sequences of the ex-type strain *P*. *antarcticum* CBS 100492^T^, whereas the ITS regions and partial *CaM* genes were more than 99% identical. Moreover, all KMM 4711-KMM 4715 had 100% sequence identity of ITS, *BenA*, and *CaM* with those of the original strain KMM 4668. The phylogenetic ML tree of the concatenated ITS-*BenA*-*CaM* gene sequences clearly showed that the strain KMM 4668 and its intra-strain variants cluster with the ex-type strain *P. antarcticum* CBS 100492^T^ (Figure 2).

### 3.3. PCA of UHPLC MS Chromatogramms of Fungal Extracts

To study the differences in the secondary metabolite profiles of the *P. antarcticum* strains, the extracts of KMM 4668 (Pa1), KMM 4711 (Pa2), KMM 4712 (Pa3), KMM 4713 (Pa4), KMM 4714 (Pa5), and KMM 4715 (Pa6) fungal cultures were prepared and analyzed using UPLC MS.

**Figure 3:**
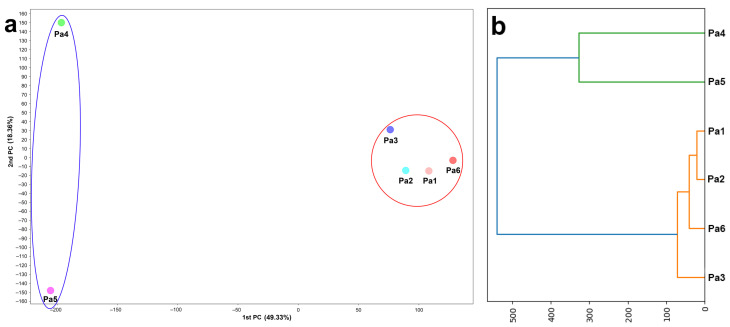
Principal component analysis plot (**a**) and dendrogram (**b**) of UPLC-MS data of the extracts of *Penicillium antarcticum* KMM 4668 (Pa1), KMM 4711 (Pa2), KMM 4712 (Pa3), KMM 4713 (Pa4), KMM 4714 (Pa5), and KMM 4715 (Pa6).

To show the statistical differences between the extracts, a PCA and a dendrogram were used. The optimal number of principal components (PCs) turned out to be two; therefore, two PCs (PC1 and PC2) were chosen to describe 67.69% of the variations in the samples. The PC1 describes 49.33% of the variance, and the PC2 describes 18.36% of the variations (Figure 3).

Extracts Pa1–Pa3 and Pa6 showed minimal differences between them in both components and were located in the same cluster. At the same time, the Pa4 and Pa5 extracts differed only in the second component and differed in the first component with the first cluster.

The dendrogram confirmed the relationships between the extracts visualized on the PCA plot, showing maximum similarity between the Pa4 and Pa5 extracts.

### 3.4. Bioassay

The influence of fungal extracts on the viability of four mammalian cell lines was investigated, and the data are presented in Table 1.

The extracts Pa1, Pa2, Pa3, and Pa6 decreased the viability of HeLa cells by 90.9–91.2%, PC-3 cells by 80.1–86.7%, MCF-7 cell by 87.3–87.9%, and H9c2 cells by 77.1–80.7%. At the same time, Pa4 and Pa5 extracts decreased the viability of HeLa cells by 66.9% and 72.6%, PC-3 cells by 37.7% and 27.2%, MCF-7 cells by 63.8% and 68.5%, and H9c2 cells by 68.4% and 58.1%, respectively.

### 3.5. Identification of the Compounds in UHPLC MS Chromatogramms of P. antarcticum Strains

**Figure 4:**
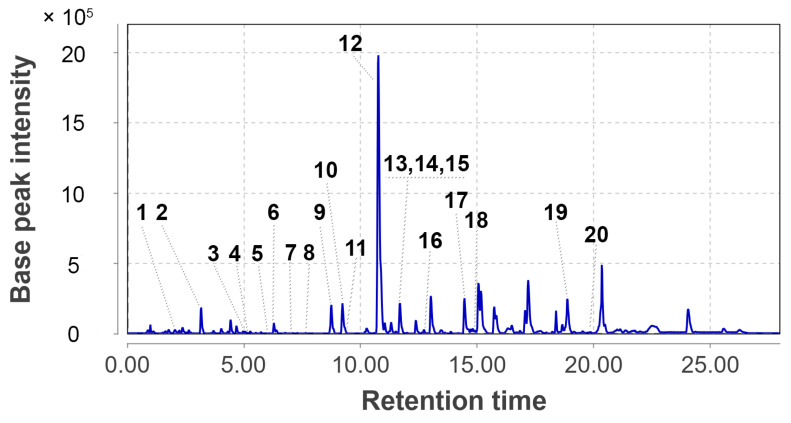
UPLC MS chromatogram of *Penicillium antarcticum* KMM 4668 extract.

The UPLC MS chromatogram of the *P. antarcticum* KMM 4668 culture extract (Pa1) is shown in Figure 4.

The GNPS database, MetFrag service, and in-house database were used to annotate the low-molecular-weight compounds in the extract.

Peak #2, detected at 3.15 min with *m*/*z* 191.0805, corresponded with the molecular formula C_10_H_10_N_2_O_2_, the same as chrysogine, which was previously isolated from the fungus *Penicillium chrysogenum* [15]. The peak was associated with the compound using the GNPS database (MQScore 0.87).

Peak #3, detected at 5.05 min (*m*/*z* 325.1285), corresponded with the molecular formula C_16_H_20_O_7_, the same as 14-hydroxyasperentin B, which was previously isolated from the fungus *P. antarcticum* KMM 4685 [17]. The compound was identified using an in-house database.

The peaks #4 and #5, detected at 5.18 and 6.08 min, respectively, with *m*/*z* 283.1552, corresponded to the molecular formula C_15_H_22_O_5_. These peaks were suggested as piltunine A, piltunine C, and penigrisacid D, which were previously isolated from this strain [20].

Peak #12, detected at 10.76 min at *m*/*z* 293.1377, corresponded with the molecular formula C_16_H_20_O_5_, and was assigned to cladosporin, the usual polyketide of *P*. *antarcticum*. The compound was identified based on its exact mass value and RT using an in-house database. The peaks #6 and #10, detected at 6.28 and 9.23 min at *m*/*z* 309.1332, corresponded with the molecular formula C_16_H_20_O_6_. These peaks showed characteristic MS/MS spectra for the monohydroxylated derivative of cladosporin, such as cladomarin and 5′-hydroxyasperentin, which were isolated from the fungus of this species.

The peaks #7 and #8, detected at 7.12 and 7.37 min with *m*/*z* 267.1227, corresponded with the molecular formula C_14_H_18_O_5_ that can be annotated with piltunins E and F, metabolites of this strain.

Peak #11, detected at 9.32 min with *m*/*z* 267.1585, corresponded with the molecular formula C_15_H_22_O_4_ that can be annotated with aspterric acid, a possible biosynthetic precursor of piltunins.

Peak #13, detected at 11.69 min with *m*/*z* 501.2126, corresponded with the molecular formula C_27_H_32_O_9_, which was the same as austin, the characteristic meroterpenoid of *P*. *antarcticum*. The compound was identified using the GNPS database (MQScore 0.95).

Peak #14, detected at 11.69 min with *m*/*z* 523.2281, corresponded with the molecular formula C_28_H_36_O_8_, the same as citreohybridonol, a common androstane-type meroterpenoid of *Penicillium* fungi. The compound was identified using the GNPS database (MQScore 0.95). Peak #15, detected at 11.69 min with *m*/*z* 441.2272, corresponded with the molecular formula C_26_H_32_O_6_, the same as tropolactone C, a widespread fungal meroterpenoid. The compound was annotated based on the exact mass value and MetFrag service.

Peak #16 was detected at 12.72 min with *m*/*z* 318.2789, corresponding to the molecular formula C_21_H_35_NO, the same as preussin, a hydroxyl pyrrolidine derivative from the sponge-associated fungus *Aspergillus candidus*. The compound was identified using the GNPS database (MQScore 0.81).

Peak #17 was detected at 14.48 min with *m*/*z* 507.2282, corresponding to the molecular formula C_32_H_30_N_2_O_4_, the same as asperphenamate, a linear amino acid ester from fungi *P. astrolabium*. The compound was identified using the GNPS database (MQScore 0.91).

Peak #18 was detected at 14.92 min with *m*/*z* 279.2320, corresponding to the molecular formula C_18_H_30_O_2_. The peak was suggested to be an unidentified fatty acid with four degrees of unsaturation based on tandem MS data and exact mass.

Peak #19 was detected at 18.87 min with *m*/*z* 282.2793, corresponding to the molecular formula C_18_H_35_NO. The compound was annotated as an amide of octadecenoic fatty acid based on its exact mass and fragmentation pattern.

Peak #20 was detected at 19.85 min with *m*/*z* 411.3259, corresponding to the molecular formula C_28_H_44_O_3_, the same as ergosterol peroxide, a usual derivative of the main fungal triterpenoid ergosterol. The compound was suggested based on an exact mass value and RT with an in-house database, and using the GNPS database (MQScore 0.82).

Peak #1, detected at 2.03 min (*m*/*z* 195.1008) based on the fragmentation pattern, was assumed to be an unknown polyketide derivative with the molecular formula C_11_H_14_O_3_. Peak #9, detected at 8.75 min (*m*/*z* 535.2716), can be assigned to an unknown alkaloid with the molecular formula C_33_H_34_N_4_O_3_.

In total, 20 compounds were annotated using the GNPS database, the in-house database, or the MetFrag service. Detailed characteristics of the identified compounds are presented in the Appendix A (Table A1).

It should be noted that we have not been able to find convincing evidence for the production of cyclopiane diterpenes that are characteristic of another *P. antarcticum* strain [19].

**Figure 5:**
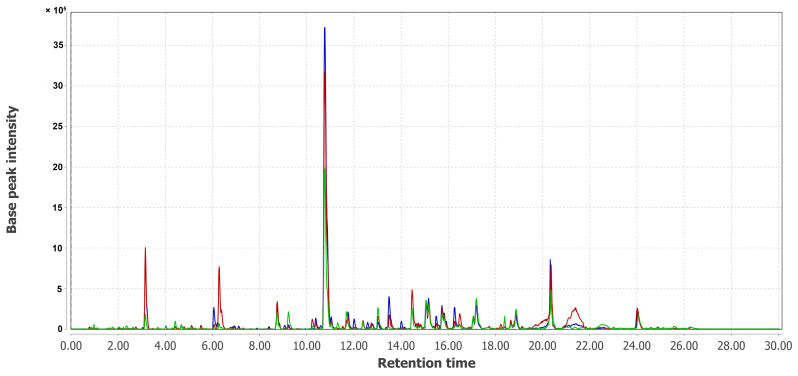
UPLC-MS chromatogram comparison of extracts of KMM 4668 (Pa1, green) and its intra-strain variants (Pa4, blue and Pa5, red).

**Figure 6:**
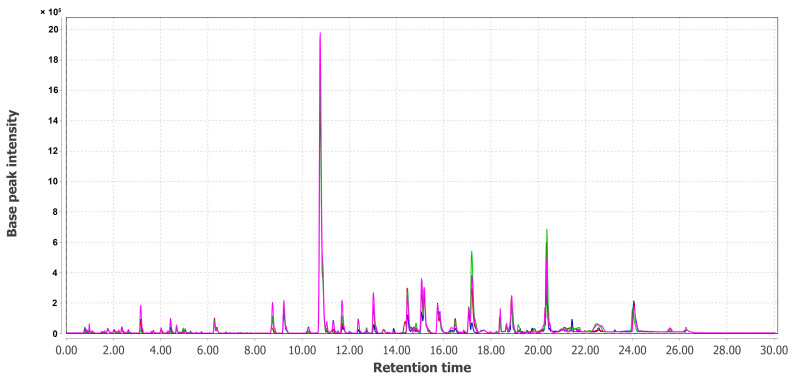
UPLC-MS chromatogram comparison of extracts of KMM 4668 (Pa1, magenta) and its intra-strain variants (Pa2, green, Pa3, red and Pa6, blue).

The combined UPLC MS chromatograms of KMM 4668 (Pa1), KMM 4711 (Pa2), KMM 4712 (Pa3), KMM 4713 (Pa4), KMM 4714 (Pa5), and KMM 4715 (Pa6) extracts are presented in Figure 5 and Figure 6.

**Figure 7:**
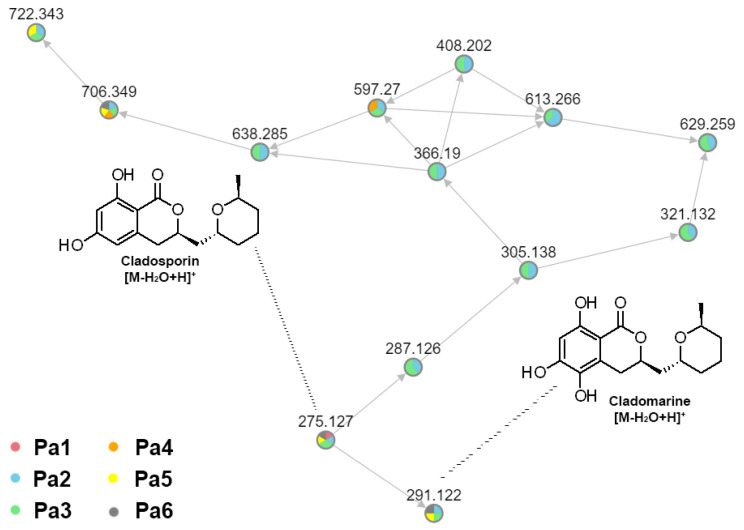
Feature-based molecular network (FBMN) of extracts of intra-strain variants of KMM 4668. Feature identity is annotated with the results from the in-house library. The precursor mass of all annotated compounds corresponds to the [M + H]^+^ adduct of these compounds. A pie chart shows the distribution of the metabolite extract according to the legend.

The use of a FBMN, together with the in-house database, made it possible to reveal a cluster of related features, which were assigned to cladosporin-related compounds. Unfortunately, we were only able to assign two compounds in the cluster, and most of the features remained unidentified (Figure 7).

**Figure 8:**
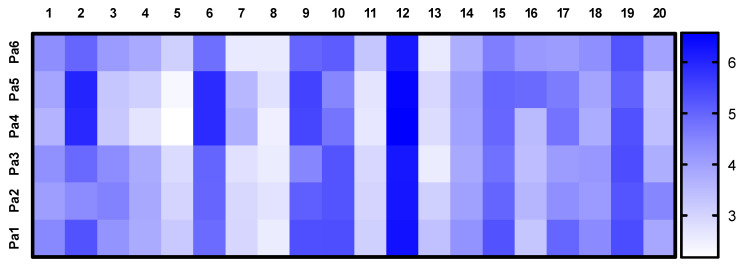
The heatmap of a related content of compounds identified in the extracts of *Penicillium antarcticum* KMM 4668 (Pa1), KMM 4711 (Pa2), KMM 4712 (Pa3), KMM 4713 (Pa4), KMM 4714 (Pa5), and KMM 4715 (Pa6). Each cell represents a decimal logarithm of the peak area in the UPLC-MS chromatogram.

The relative contents of the announced compounds calculated as a decimal logarithm of the peak area of these peaks in the UPLC-MS chromatograms of Pa1, Pa2, Pa3, Pa4, Pa5, and Pa6 extracts were visualized in the heatmap (Figure 8). Cladosporin (#12) was the main component of each extract. Extracts Pa4 and Pa5 were different from other extracts, with a large content of chrysogine (#2) and an unidentified cladosporin derivative (#6).

## 4. Discussion

Phenotypic diversity is one of the ways that fungi adapt to changing environments. Three types of phenotypic changes have been reported: morphological transitions (MT) induced by environmental signals, phenotypic switching (PS) that occurs in a small fraction of the population and is not necessarily induced by external signals, and antigenic variation (AV), which involves alternating the expression of surface proteins (or carbohydrates) [30]. Phenotypic switching is associated with metabolic changes [31], which are a part of the environmental adaptation of microorganisms. For example, aflatoxin production by *Aspergillus flavus* was reduced over several generations of laboratory fermentation owing to the absence of natural competition, and was recovered in co-cultivation with other fungi [32].

Various strains of a microbial species may exhibit different phenotypic variations. Using single-cell measurements, the phenotypic diversity of 37 natural strains of *Saccharomyces cerevisiae* yeasts was investigated, and these wild strains displayed different levels of noise in single-cell traits [33]. This suggests a different level of phenotypic diversity in different fungal strains, which can lead to the isolation of single-cell isolates with different secondary metabolites [21]. The biosynthesis of secondary metabolites is controlled by biosynthetic gene clusters (BGCs), and changes in these clusters can result in a change in compound yield [34]. From an evolutionary perspective, new metabolic capabilities typically emerge through some form of gene duplication, acquisition of novel genes through horizontal gene transfer, and, in addition, novelty might emerge through the establishment of a new regulatory network [35].

It is known that the phenotypic diversity of fungi, expressed both at the level of morphology and production of secondary metabolites, is due to the phenomenon of heterokaryosis, which provides haploid organisms with significant adaptive advantages [36]. Heterocaryosis is of great importance to fungi, whose life cycle is exclusively represented by the anamorphic stage [36]. Most fungi, including *Penicillium* species, primarily exist as heterokaryons in nature. They are more flexible and viable than homokaryons, which play a significant role in the colonization of a variety of natural substrates [37]. However, the heterokaryotic state of most fungi that reproduce via mononuclear conidia is unstable, and when such conidia are dispersed, the heterokaryon disintegrates easily into the original homokaryons. Different phenotypic variants representing the original parental forms were isolated during a study of the KMM 4668 strain. The latter differed not only in terms of cultural and morphological characteristics, but also in the ability to produce biologically active compounds.

In our study, we observed differences in chrysogine and cladosporin-related compounds, as well as sesquiterpenes piltunin production in KMM 4713 and KMM 4714 intra-strain variants of *P. antarcticum* KMM 4668. The biosynthesis of cladosporin and related compounds is well known and carried out under the sequential action of two type I polyketide synthases [38]. It was found earlier that biosynthesis of chrysogine is carried out by gene clusters from seven genes [15]. Piltunins are the closest derivatives of the well-known herbicide carotanoid aspterric acid. In particular, piltunins A and C are products of the Asp hydroxylation of aspterric acid. It is known that biosynthesis of aspterric acid is controlled by a BGC, including genes encoding a sesquiterpene cyclase, two P450 monooxygenases, and the self-resistance gene *astD* [39]. The production of these compounds was enhanced in the KMM 4713 and KMM 4714 strains, which may be the result of changes in the described BGCs. However, further investigations are required.

At the same time, the cytotoxic effects of the KMM 4713 and KMM 4714 extracts were lower than those of the other extracts (Table 1), which may be the result of a decrease in the concentration of cytotoxic components or an increase in cytoprotective compounds. As shown in the heatmap (Figure 8), the contents of #3 (14-hydroxyasperentin B), #4–5 (piltunine A, piltunine C, and penigrisacid D), #10 (cladosporin derivative), and #18 (unsaturated fatty acid) were lower in the KMM 4713 and KMM 4714 extracts. However, the cytotoxic activity of 14-hydroxyasperentin B has not been studied. Piltunine A, piltunine C, and penigrisacid D showed weak cytotoxic activity against prostate cancer 22Rv1 cells and normal prostate PNT-2 cells, and anti-inflammatory activity in LPS-stimulated RAW264.7 macrophages [20]. Cladosporin and its derivatives have various bioactivities, including potent antimalarial activity [40,41].

Based on the outcome of our research, the intra-strain variants of *P. antarcticum* may be a promising source of bioactive secondary metabolites.

## 5. Conclusions

The intra-strain variants of *P. antarcticum* KMM 4668 were isolated and labelled KMM 4711, KMM 4712, KMM 4713, KMM 4714, and KMM 4715. The investigation of molecular markers such as ITS regions, partial *BenA*, *CaM*, and *RPB2* gene sequences indicated that KMM 4668 belongs to *P. antarcticum*. A comparison of the first three phylogenetic makers between the original strain KMM 4668 and its variants KMM 4711–KMM 4715 showed their 100% identity. Differences in the UPLC MS metabolite profiles of these variants were observed in cladosporin, chrysogine, and piltunin production. The variants KMM 4713 and KMM 4714 were characterized by increased production of most of the cladosporin-related compounds, alkaloid chrysogine, and piltunin sesquiterpenoids. Thus, these variants could be used to obtain these metabolites.

## Figures and Tables

**Table 1:** The cytotoxic activity of the *Penicillium antarcticum* extracts.

Extracts	Cell Viability, % of Control			
	HeLa	PC-3	MCF-7	H9c2
Pa1	8.8 ± 0.1	17.1 ± 4.6	12.1 ± 0.1	19.4 ± 0.6
Pa2	8.7 ± 0.4	13.9 ± 3.9	12.7 ± 0.6	20.7 ± 4.4
Pa3	9.1 ± 0.5	13.3 ± 1.0	12.5 ± 0.2	19.3 ± 1.0
Pa4	33.1 ± 3.4	62.3 ± 2.4	36.2 ± 2.9	31.6 ± 1.5
Pa5	27.4 ± 3.6	72.8 ± 2.7	31.5 ± 0.6	41.9 ± 0.7
Pa6	8.9 ± 0.1	19.9 ± 0.9	12.5 ± 0.6	22.9 ± 0.9

*Penicillium antarcticum* KMM 4668 (Pa1), KMM 4711 (Pa2), KMM 4712 (Pa3), KMM 4713 (Pa4), KMM 4714 (Pa5), and KMM 4715 (Pa6). All extracts were used at a concentration of 10 mkg/mL. The assays were carried out in triplicate and the data are presented as the mean ± standard error of mean.

## Data Availability

The original contributions presented in this study are included in the article and Supplementary Material. Further inquiries can be directed to the corresponding authors.

## Supplementary Materials

The following supporting information can be downloaded at https://www.mdpi.com/article/10.3390/metabo15020077/s1, Figure S1: HR (+)ESI MS/MS spectrum of 1; Figure S2: HR (+)ESI MS/MS spectrum of 2; Figure S3: HR (+)ESI MS/MS spectrum of 3; Figure S4: HR (+)ESI MS/MS spectrum of 4; Figure S5: HR (+)ESI MS/MS spectrum of 6; Figure S6: UPLC MS chromatogram of *Penicillium antarcticum* KMM 4668 extract (Pa1); Figure S7: UPLC MS chromatogram of *Penicillium antarcticum* KMM 4711 extract (Pa2); Figure S8: UPLC MS chromatogram of *Penicillium antarcticum* KMM 4712 extract (Pa3); Figure S9: UPLC MS chromatogram of *Penicillium antarcticum* KMM 4713 extract (Pa4); Figure S10: UPLC MS chromatogram of *Penicillium antarcticum* KMM 4714 extract (Pa5); Figure S11: UPLC MS chromatogram of *Penicillium antarcticum* KMM 4715 extract (Pa6); Table S1: The strains used in multi-locus phylogenetic analysis and GenBank accession numbers; Table S2: Amounts of the extracts of the fungal cultures; Supplementary Text: UHPLC-Q-TOF Data Analysis. References [42,43,44,45,46] are cited in the Supplementary Materials.

## Appendix A

**Table A1:** The list of annotated compounds.

#	RT, min	Measured *m*/*z*	MF	Calculated *m*/*z*	Error, ppm	MQScore (GNPS)	Structure	Name and Identification Level	Ref.
1	2.03	195.1008 [M + H]^+^	C_11_H_14_O_3_	195.1016	−3.95	-	-	-	-
2	3.15	191.0805 [M + H]^+^	C_10_H_10_N_2_O_2_	191.0815	−5.25	0.87		Chrysogine ^b^	[15]
3	5.05	325.1285 [M + H]^+^	C_16_H_20_O_7_	325.1282	0.99	-		14-hydroxyasperentin B ^a^	[17]
4	5.18	283.1552 [M + H]^+^	C_15_H_22_O_5_	283.1540	4.24	-		Penigrisacid D ^c^	[47]
						-		Piltunine C ^c^	[20]
5	6.08	283.1552 [M + H]^+^	C_15_H_22_O_5_	283.1540	4.24	-		Piltunine A ^c^	[20]
6	6.28	309.1332 [M + H]^+^	C_16_H_20_O_6_	309.1333	−0.21	-		Hydroxyasperentin B ^c^ (Cladomarin)	[8]
						-		14-hydroxyasperentin ^c^ (14-hydroxycladosporin)	[17]
						-		13-hydroxyasperentin ^c^ (13-hydroxycladosporin)	[48]
7	7.12	267.1227 [M + H]^+^	C_14_H_18_O_5_	267.1227	0.00	-		Piltunine E ^c^	[20]
8	7.37	267.1227 [M + H]^+^	C_14_H_18_O_5_	267.1227	0.00	-		Piltunine F ^c^	[20]
9	8.75	535.2716 [M + H]^+^	C_33_H_34_N_4_O_3_	535.2704	2.24	-	-		
10	9.23	309.1336 [M + H]^+^	C_16_H_20_O_6_	309.1333	1.08	-		Cladomarin ^c^	[8]
						-		14-hydroxyasperentin ^c^ (14-hydroxycladosporin)	[17]
						-		13-hydroxyasperentin ^c^ (13-hydroxycladosporin)	[48]
11	9.32	267.1585 [M + H]^+^	C_15_H_22_O_4_	267.1591	−2.25	-		Aspterric acid ^c^	-
12	10.76	293.1377 [M + H]^+^	C_16_H_20_O_5_	293.1384	−2.39	-		Cladosporin ^a^ (Asperentin)	[19]
13	11.69	501.2126 [M + H]^+^	C_27_H_32_O_9_	501.2119	1.38	0.95		Austine ^b^	[49]
14	11.69	523.2281 [M + Na]^+^	C_28_H_36_O_8_	523.2302	−4.09	0.95		Citreohybridonol ^b^	[16]
15	11.69	441.2272 [M + H]^+^	C_26_H_32_O_6_	441.2272	0.08	-		Tropolactone C ^c^	[50]
16	12.72	318.2789 [M + H]^+^	C_21_H_35_NO	318.2791	−0.76	0.81		Preussin ^b^	[51]
17	14.48	507.2282 [M + H]^+^	C_32_H_30_N_2_O_4_	507.2278	0.72	0.91		Asperphenamate ^b^	[52]
18	14.92	279.2320 [M + H]^+^	C_18_H_30_O_2_	279.2319	0.51	-	-		-
19	18.87	282.2793 [M + H]^+^	C_18_H_35_NO	282.2791	0.56	-	-		-
20	19.85	411.3259 [M–H_2_O + H]^+^	C_28_H_44_O_3_	411.3258	0.35	0.82		Ergosterol peroxide ^a^	[53]

^a^—identification was made by comparing the RT, MS, and MS/MS data with those of authentic standards; ^b^—structure was annotated by comparing the obtained MS/MS data with available data in the in-house database or in the GNPS; ^c^—structure was assigned based on MF and literature data.
