# Supplementary material for: Differences in Metabolite Profiles and Bioactivities of Intra-Strain Variants of Marine Fungus Penicillium antarcticum KMM 4668
Source: Metabolites. 2025 Jan 27;15(2):77. doi: 10.3390/metabo15020077 (PMC11857625; doi:10.3390/metabo15020077)
Supplement: Supplementary file 1 [file metabolites-15-00077-s001.zip › metabolites-3371161-supplementary.pdf]

SUPPLEMENTARY MATERIAL

Differences in metabolite profiles and bioactivities of intra-strain variants of marine fungus *Penicillium antarcticum* KMM 4668

Content

Figure S1. HR (+)ESI MS/MS spectrum of 1 5
Figure S2. HR (+)ESI MS/MS spectrum of 2 6
Figure S3. HR (+)ESI MS/MS spectrum of 3 7
Figure S4. HR (+)ESI MS/MS spectrum of 4 8
Figure S5. HR (+)ESI MS/MS spectrum of 6 9
Figure S6. UPLC MS chromatogram of *Penicillium antarcticum* KMM 4668 extract (Pa1). 10
Figure S7. UPLC MS chromatogram of *Penicillium antarcticum* KMM 4711 extract (Pa2). 11
Figure S8. UPLC MS chromatogram of *Penicillium antarcticum* KMM 4712 extract (Pa3). 12
Figure S9. UPLC MS chromatogram of *Penicillium antarcticum* KMM 4713 extract (Pa4). 13
Figure S10. UPLC MS chromatogram of *Penicillium antarcticum* KMM 4714 extract (Pa5). 14
Figure S11. UPLC MS chromatogram of *Penicillium antarcticum* KMM 4715 extract (Pa6). 15
Table S1. The strains used in multi-locus phylogenetic analysis and GenBank accession numbers. 16
Table S2. Amounts of the extracts of the fungal cultures. 17
UHPLC-Q-TOF Data Analysis 18
References 18

Figure S1. HR (+)ESI MS/MS spectrum of 1

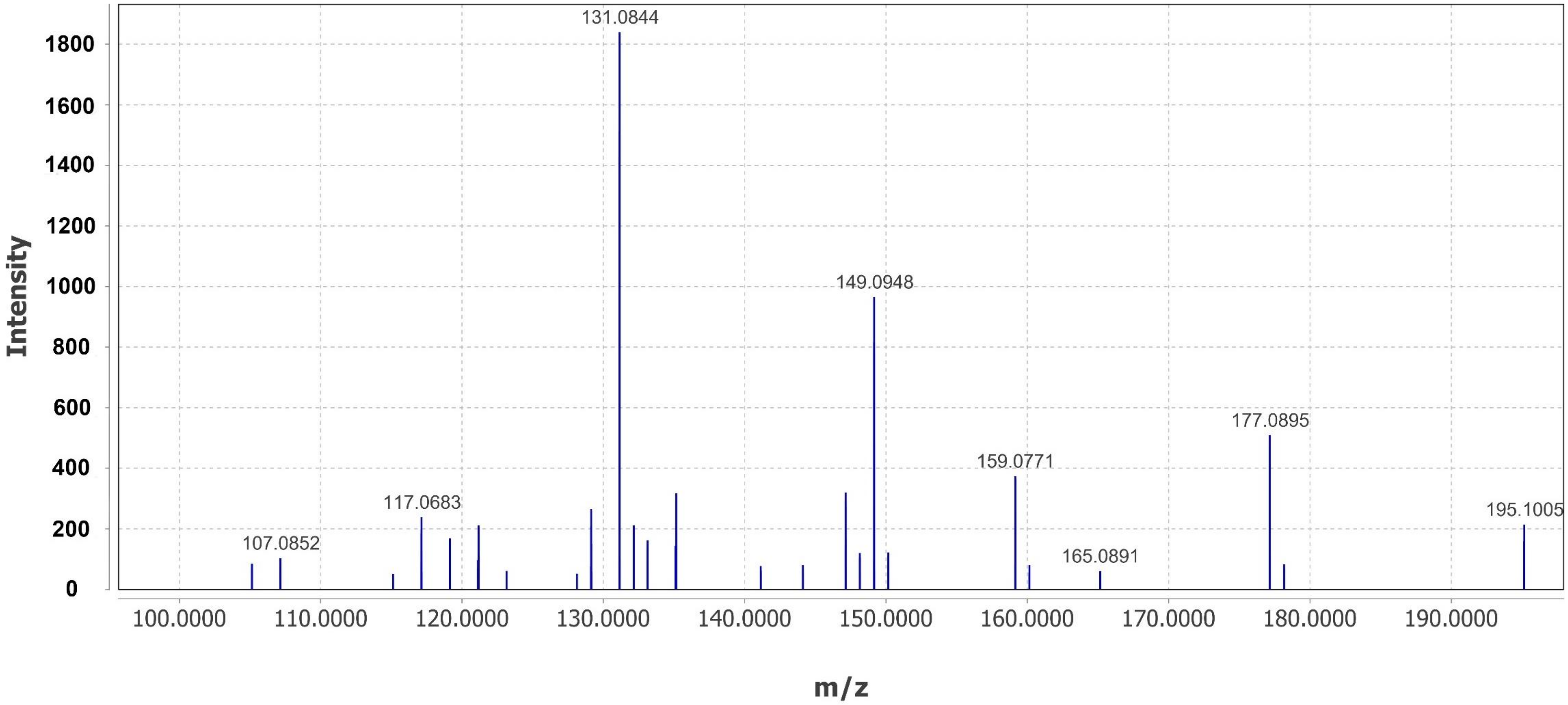

Figure S2. HR (+)ESI MS/MS spectrum of 2

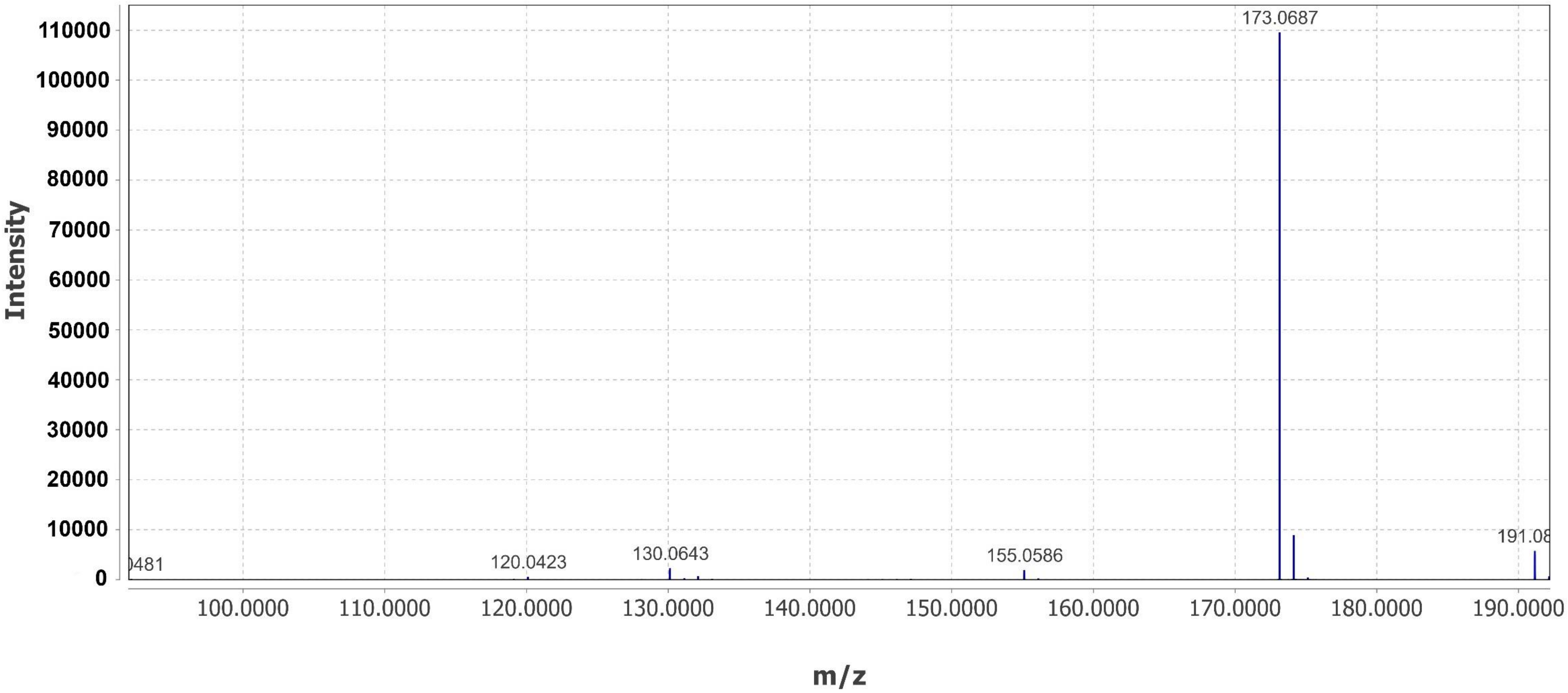

Figure S3. HR (+)ESI MS/MS spectrum of 3

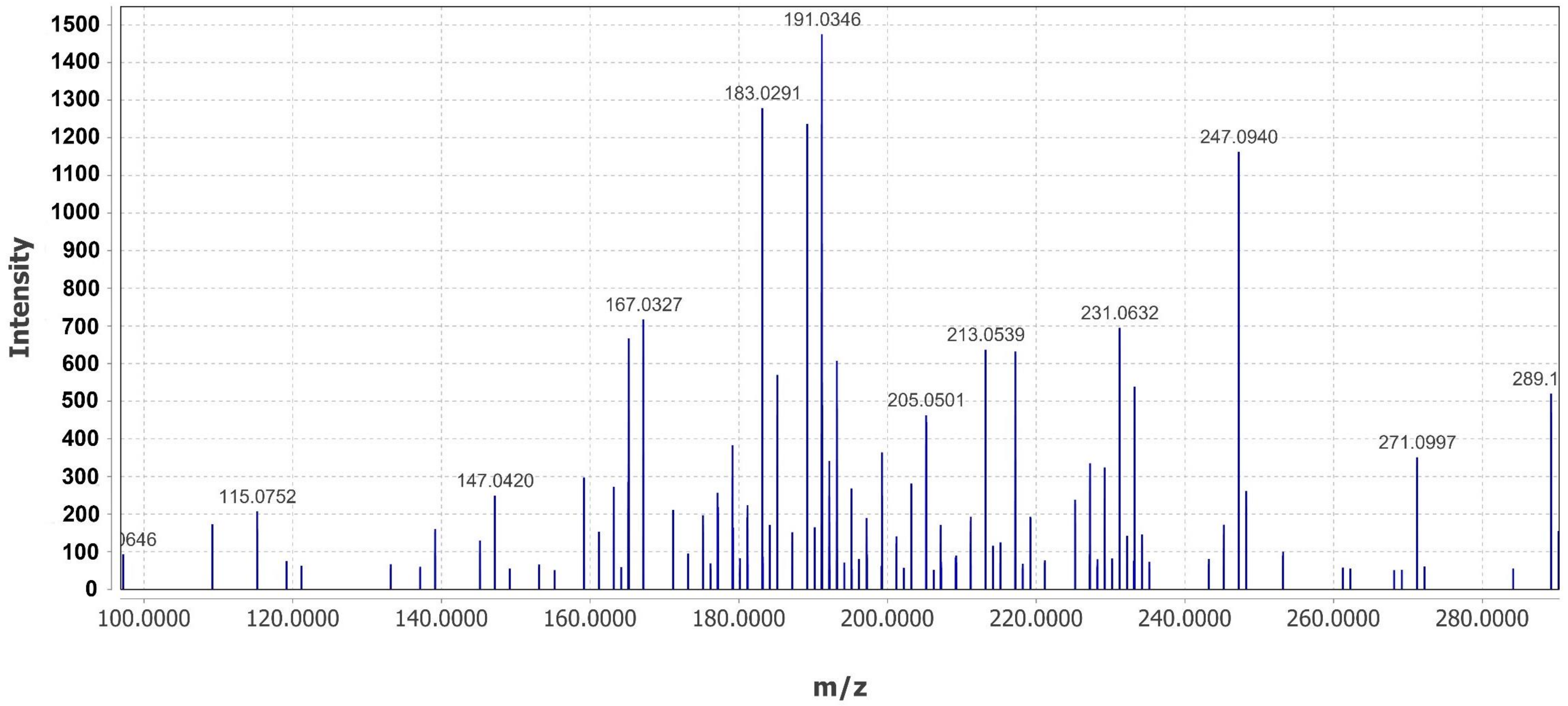

Figure S4. HR (+)ESI MS/MS spectrum of 4

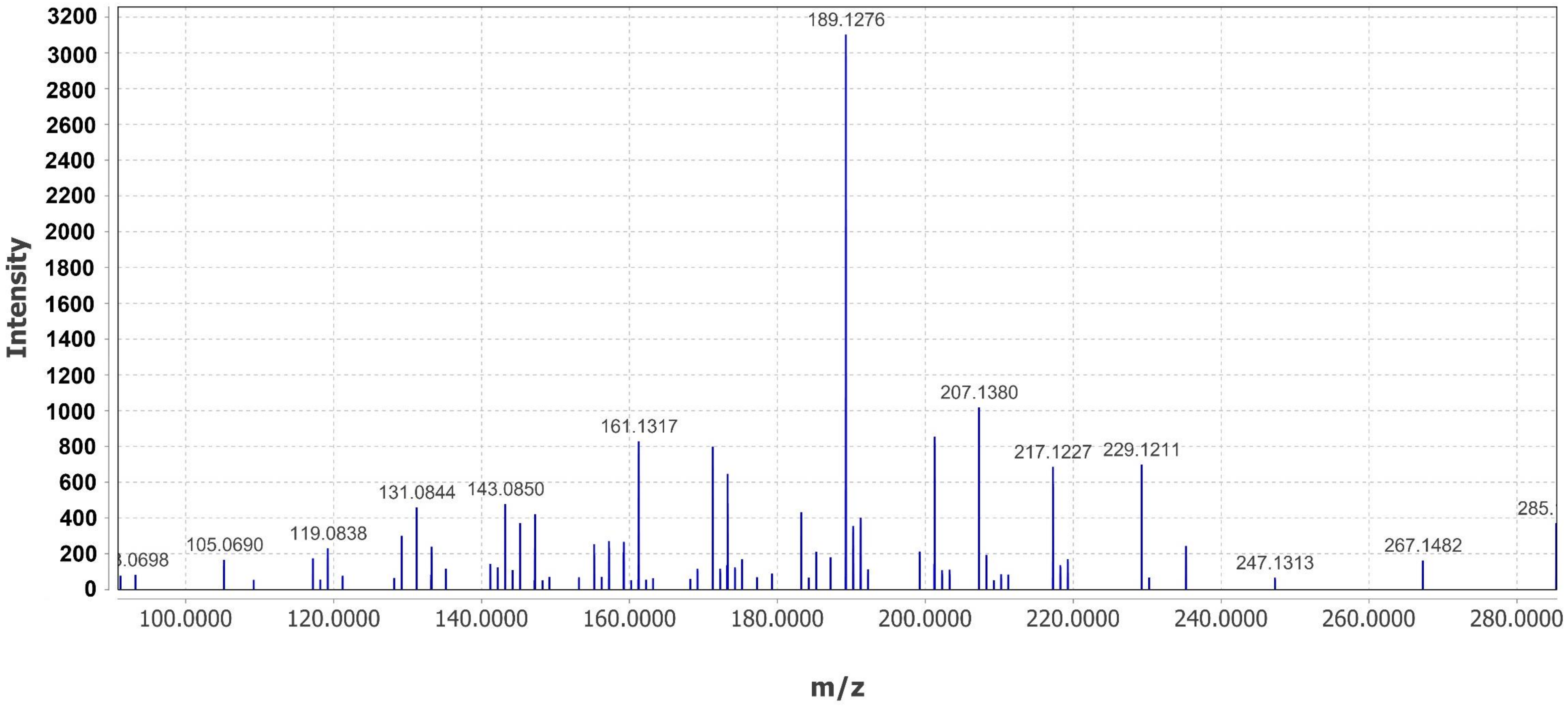

Figure S5. HR (+)ESI MS/MS spectrum of 6

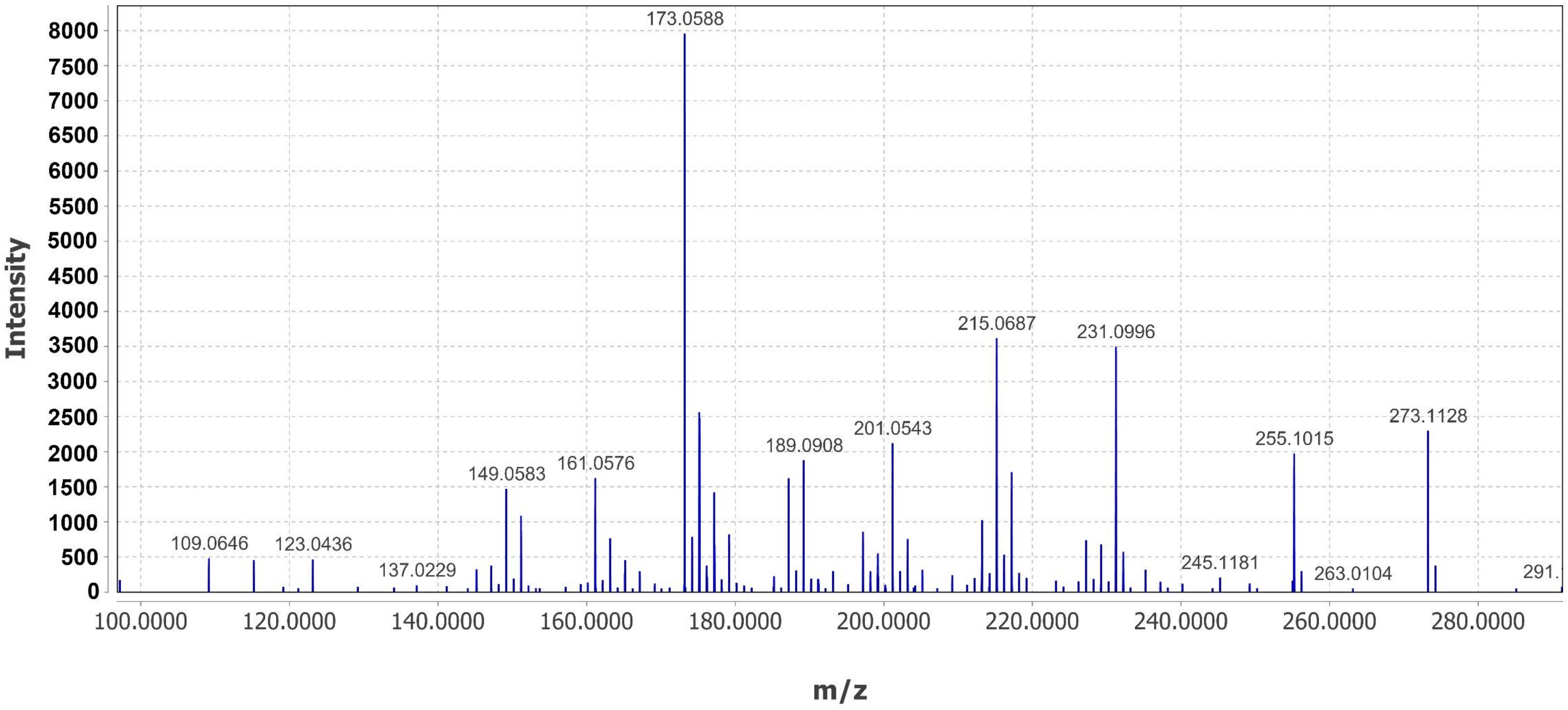

Figure S6. UPLC MS chromatogram of *Penicillium antarcticum* KMM 4668 extract (Pa1).

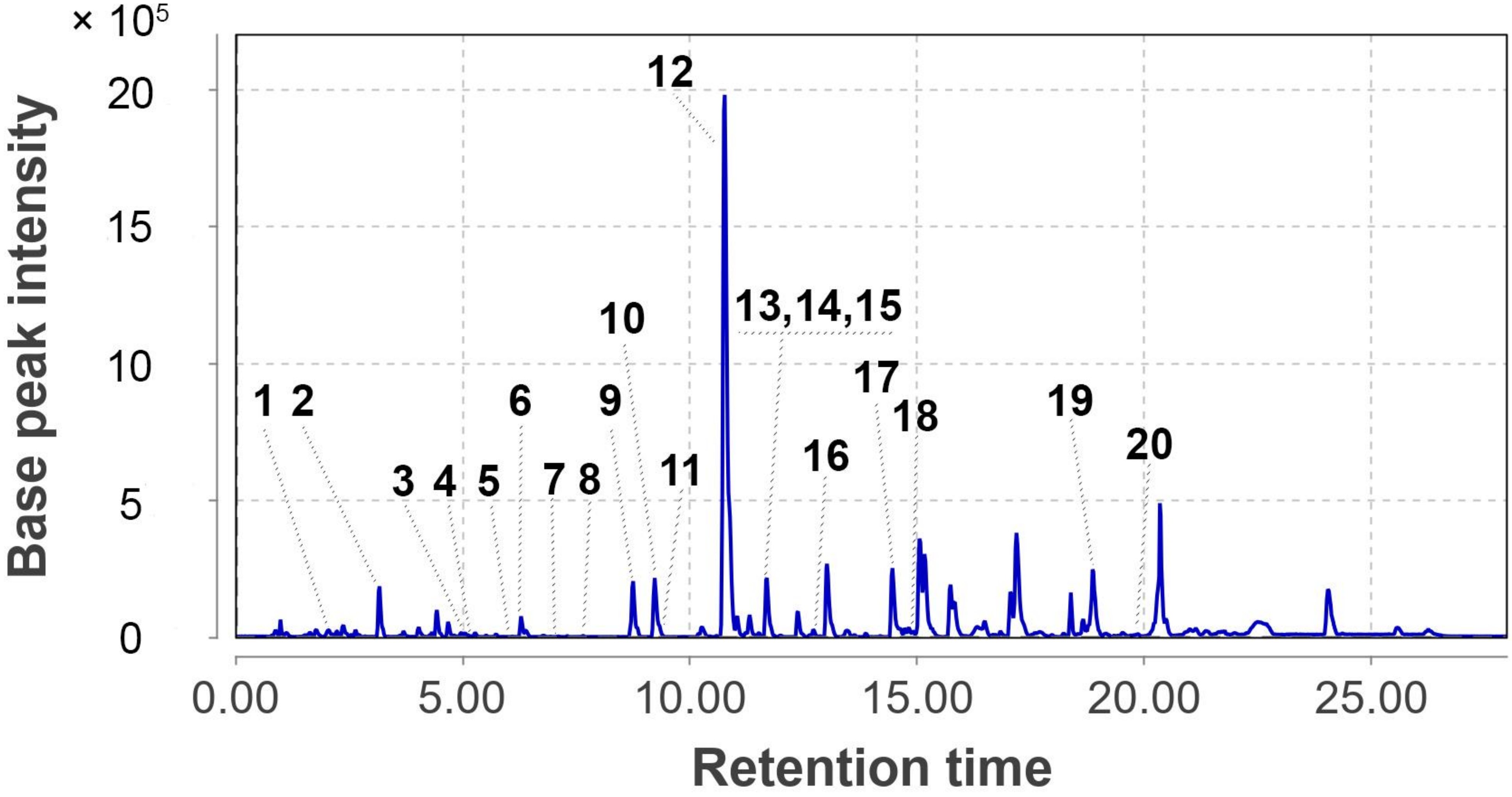

Figure S7. UPLC MS chromatogram of *Penicillium antarcticum* KMM 4711 extract (Pa2).

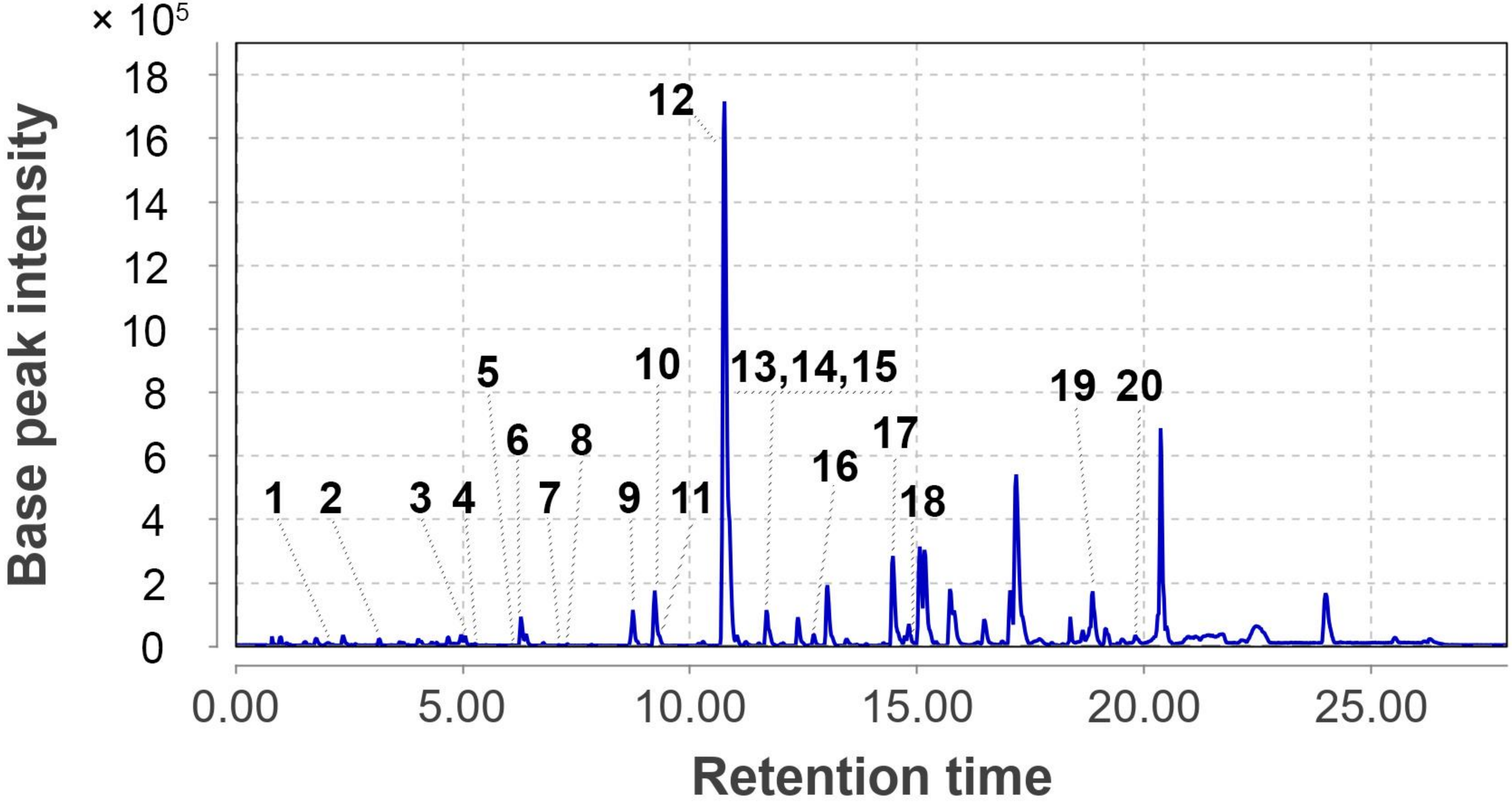

Figure S8. UPLC MS chromatogram of *Penicillium antarcticum* KMM 4712 extract (Pa3).

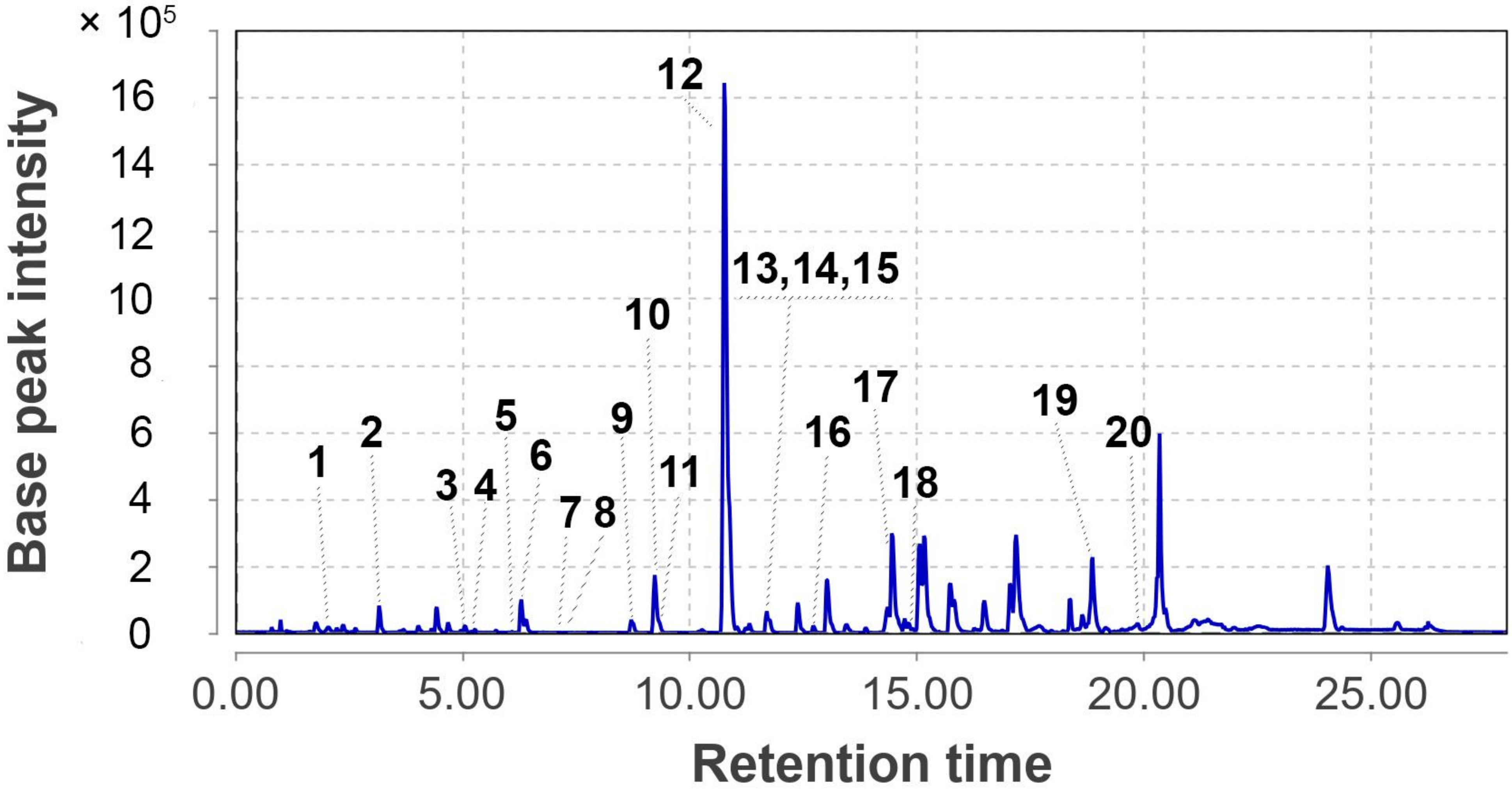

Figure S9. UPLC MS chromatogram of *Penicillium antarcticum* KMM 4713 extract (Pa4).

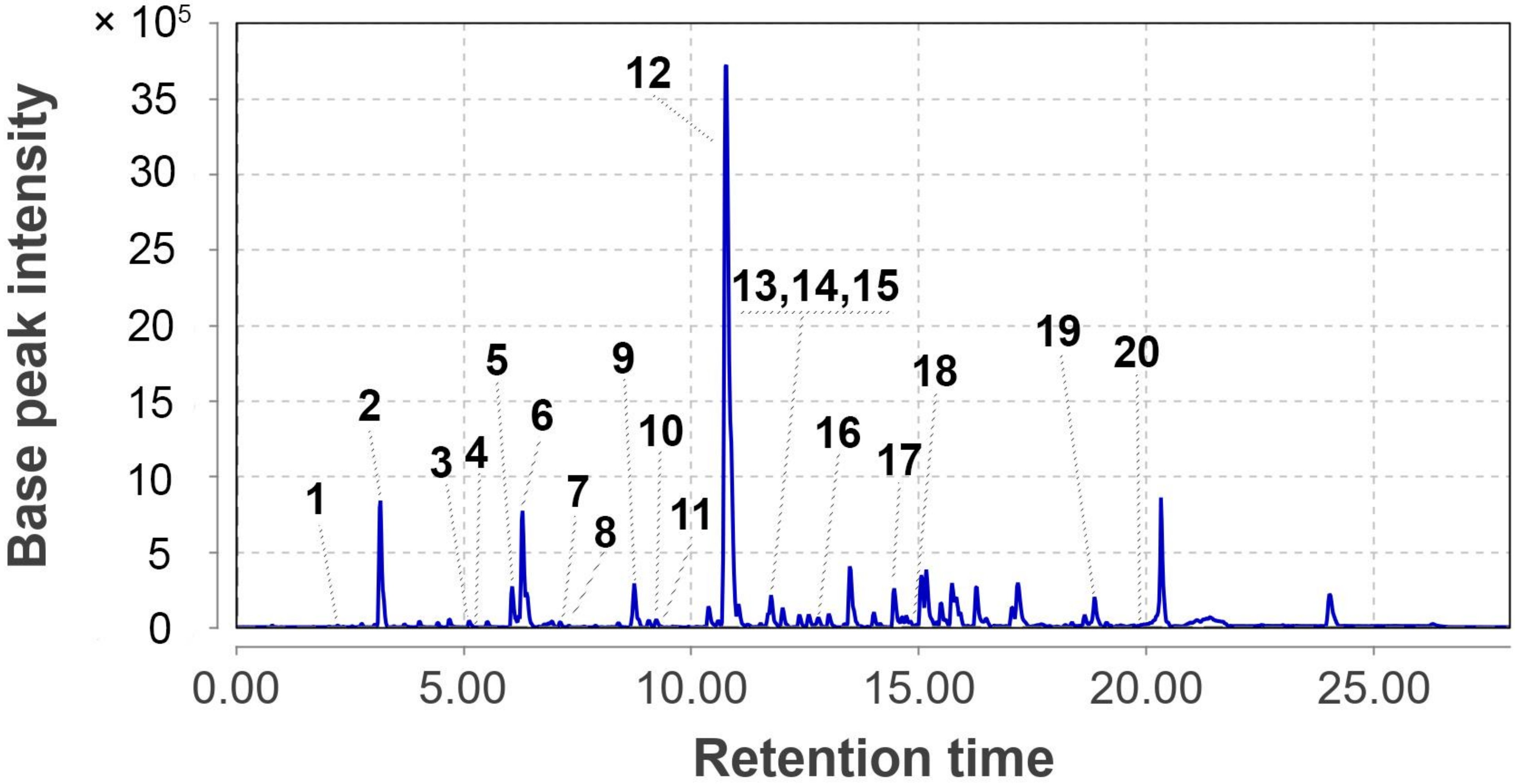

Figure S10. UPLC MS chromatogram of *Penicillium antarcticum* KMM 4714 extract (Pa5).

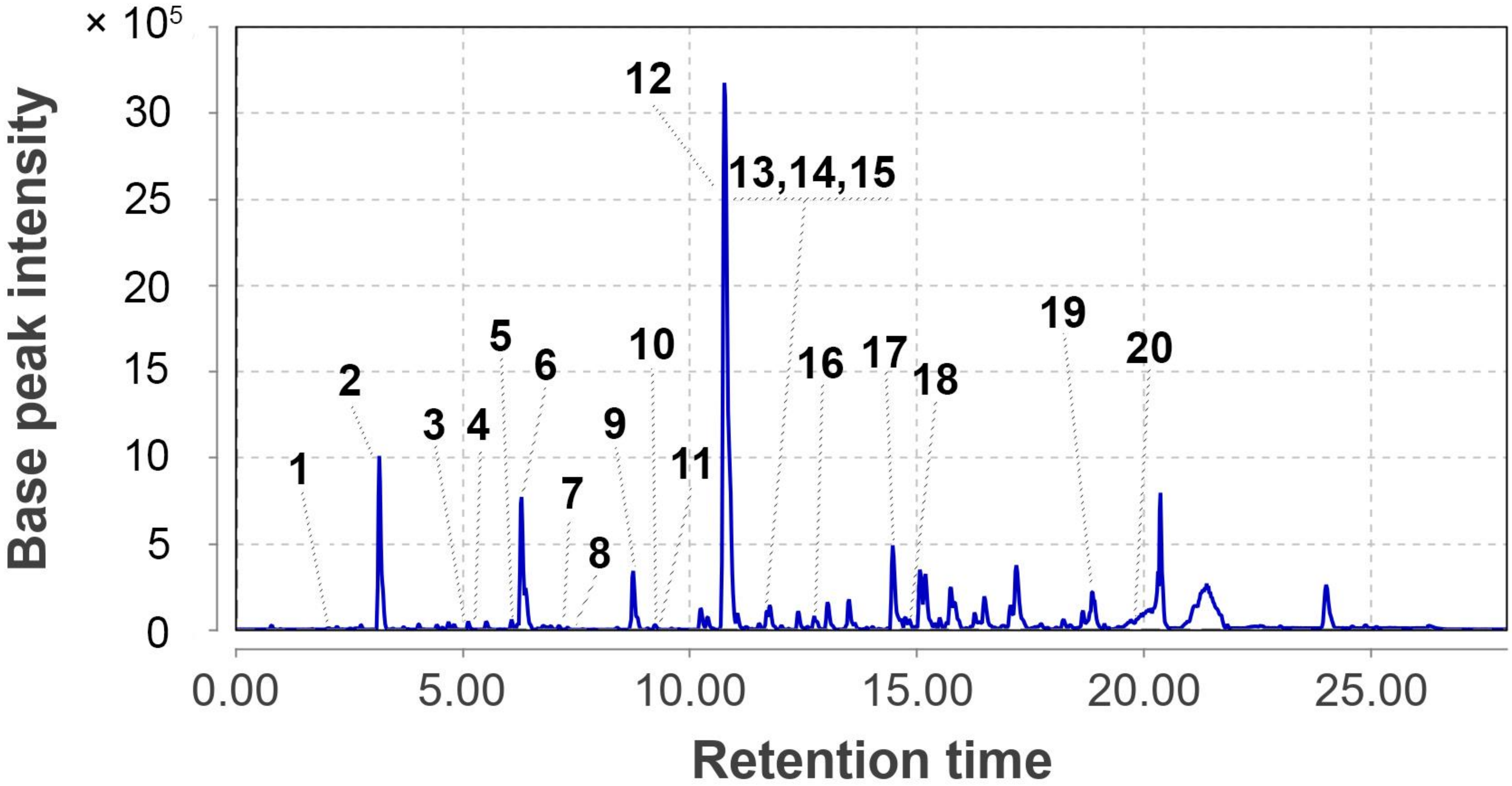

Figure S11. UPLC MS chromatogram of *Penicillium antarcticum* KMM 4715 extract (Pa6).

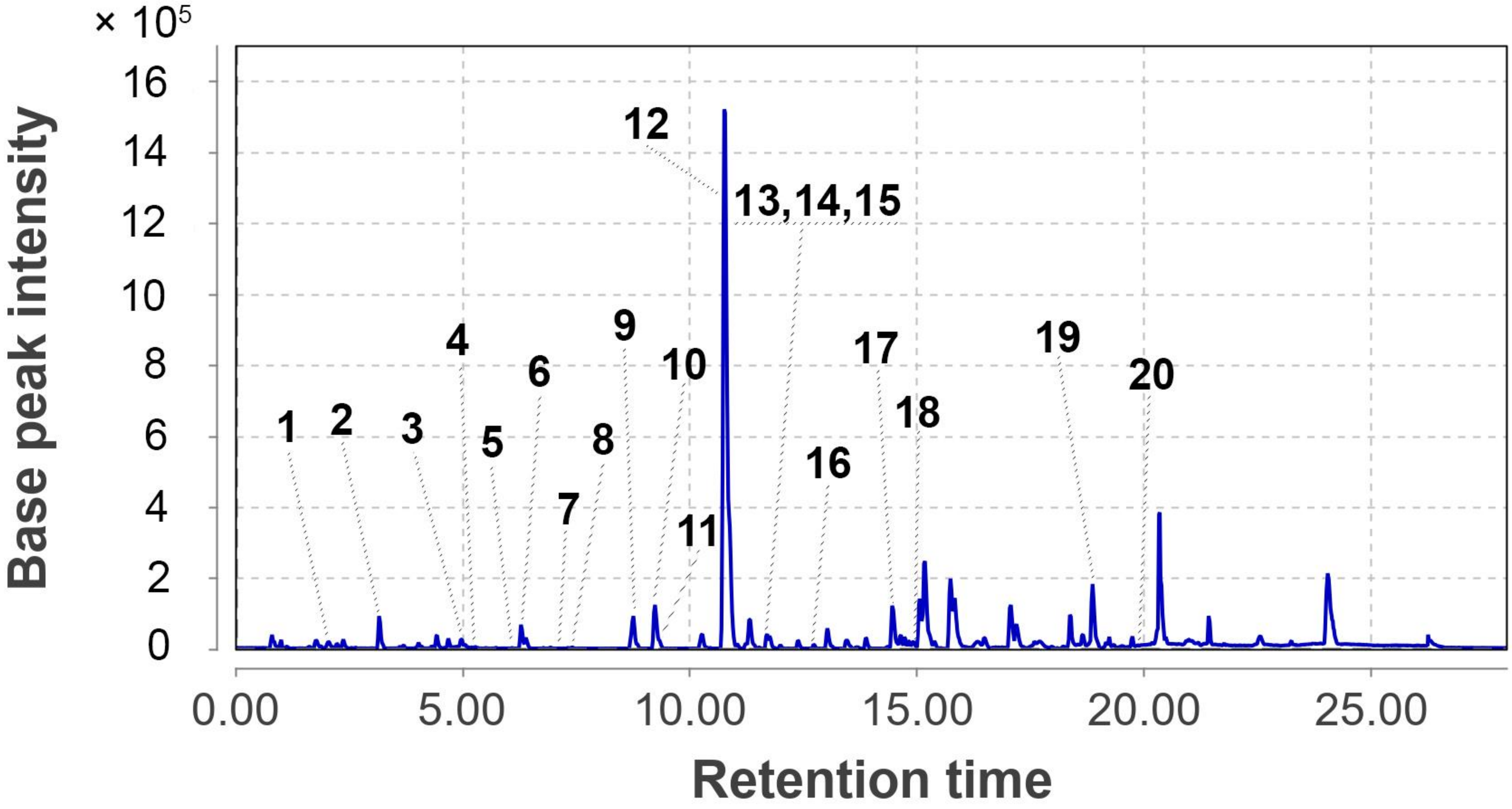

Table S1. The strains used in multi-locus phylogenetic analysis and GenBank accession numbers.

Species	Strain Number	GenBank Accession Number		
		ITS	*BenA*	*CaM*
Penicillium atrovenetum	CBS 241.56ᵀ	AF033492	JX140944	KJ867004
P. antarcticum	CBS 100492ᵀ	KJ834503	MN969371	MN969236
P. antarcticum	**KMM 4711**	**PP411239**	**PQ310240**	**PQ310245**
	**KMM 4712**	**PP411240**	**PQ310241**	**PQ310246**
	**KMM 4713**	**PP411241**	**PQ310242**	**PQ310247**
	**KMM 4714**	**PP411242**	**PQ310243**	**PQ310248**
	**KMM 4715**	**PP411243**	**PQ310244**	**PQ310249**
	**KMM 4668**	**KU358554**	**KU358557**	**KU358560**
P. doidgeae	CBS 138947ᵀ	JX140804	JX141006	JX157413
P. claroviride	CMW 56197ᵀ	MT949909	MT957414	MT957456
P. pole-evansii	CBS 138946ᵀ	JX140831	JX141005	JX157412
P. coralligerum	CBS 123.65ᵀ	JN617667	MN969378	MN969248
P. nucicola	DAOMC 250522ᵀ	KT887860	KT887821	KT887782
P. novae-zeelandiae	CBS 137.41ᵀ	JN617688	MN969390	MN969279
Talaromyces marneffei	CBS 388.87ᵀ	JN899344	JX091389	KF741958

Table S2. Amounts of the extracts of the fungal cultures.

Fungal culture	Sample code	Mass of crude extract, mg
KMM 4668 (strain)	Pa1	2.05
KMM 4711 (variant)	Pa2	1.74
KMM 4712 (variant)	Pa3	1.42
KMM 4713 (variant)	Pa4	4.85
KMM 4714 (variant)	Pa5	2.46
KMM 4715 (variant)	Pa6	3.94

UHPLC-Q-TOF Data Analysis

UHPLC-Q-TOF data were converted from Bruker ".d" formatting to ".mzXML" using MSConvert 3.0 (part of ProteoWizard 3.0 package, Palo Alto, California, USA) [49], and further processing was performed with MZMine (version 2.53) [29]. The MZMine processing settings were as follows: mass detection was carried out at the MS1 level and MS2 level with noise level thresholds of 60 and 40, respectively. Chromatograms were made with the ADAP Chromatogram Builder Module [50] with the following parameters: min group size in # of scans was set to 6, group intensity threshold and min highest intensity were set to 130 and 300, respectively, m/z tolerance was set to 0.05 m/z. The chromatogram deconvolution module was used with the ADAP algorithm with a signal/noise threshold of 8, min feature height of 300, and coefficient/area threshold of 40, while peak duration range was set from 0 to 2.0 and RT wavelet range was set from 0 to 0.1. The m/z center calculation was set to MEDIAN. The Isotopics peaks grouper module was used with an m/z tolerance of 5 ppm, retention time tolerance of 0.1 min, the monotonic shape function set to true, a maximum charge of 2, and the representative isotope set to the most intense. Alignment was achieved with the Join aligner function with an m/z tolerance of 5 ppm, a weight for m/z at 50, a retention time tolerance of 0.1 min, and a weight for RT at 50. The Require same charge state, Require same ID, and the Compare spectra similarity functions were set to false. The aligned feature list was exported using the Export/Submit to "GNPS-FBMN" module with the Merge MS/MS (experimental) function with the following parameters: select spectra to merge was set to across samples, the m/z merge mode was set to weighted average (remove outliers), the intensity merge mode was set to sum intensities, the expected mass deviation was set to 5 ppm, the cosine threshold was set to 70%, the peak count threshold was set to 20%, the isolation window offset (m/z) was set to 0, and the isolation window width (*m*/*z*) was set to 3.

Feature-based molecular networking (FBMN) was generated with the pre-processed data on the Global Natural Products Social Molecular Networking (GNPS) website (https://gnps.ucsd.edu, accessed on 21 November 2024) [51,52] and visualize as was described previously [28].

Dereplication of the MS/MS spectra was carried out using the GNPS module Library Search. All parameters were maintained as default.

Metabolite dereplication was also carried out with an in-house MS/MS spectral library, comparing experimental spectra and retention times (RT) with the spectra and RT obtained for reference compounds.

In addition, the identification of some metabolites was performed by comparing of experimental MS/MS spectra with compounds from the PubChem database using in-silica fragmentation by MetFrag service [53].

References

28. Nesterenko, L.E.; Popov, R.S.; Zhuravleva, O.I.; Kirichuk, N.N.; Chausova, V.E.; Krasnov, K.S.; Pivkin, M.V.; Yurchenko, E.A.; Isaeva, M.P.; Yurchenko, A.N. A Study of the Metabolic Profiles of *Penicillium dimorphosporum* KMM 4689 Which Led to Its Re-Identification as *Penicillium hispanicum*. *Fermentation* **2023**, *9*, 337. 10.3390/fermentation9040337
29. Pluskal, T.; Castillo, S.; Villar-Briones, A.; Orešič, M. MZmine 2: Modular framework for processing, visualizing, and analyzing mass spectrometry-based molecular profile data. *BMC Bioinform.* **2010**, *11*, 395. 10.1186/1471-2105-11-395
49. Chambers, M.C.; MacLean, B.; Burke, R.; Amodei, D.; Ruderman, D.L.; Neumann, S.; Gatto, L.; Fischer, B.; Pratt, B.; Egertson, J., *et al.* A cross-platform toolkit for mass spectrometry and proteomics. *Nat. Biotechnol.* **2012**, *30*, 918-920. 10.1038/nbt.2377
50. Myers, O.D.; Sumner, S.J.; Li, S.; Barnes, S.; Du, X. One Step Forward for Reducing False Positive and False Negative Compound Identifications from Mass Spectrometry Metabolomics Data: New Algorithms for Constructing Extracted Ion Chromatograms and Detecting Chromatographic Peaks. *Analytical Chemistry* **2017**, *89*, 8696-8703. 10.1021/acs.analchem.7b00947

51. Nothias, L.F.; Petras, D.; Schmid, R.; Dührkop, K.; Rainer, J.; Sarvepalli, A.; Protsyuk, I.; Ernst, M.; Tsugawa, H.; Fleischauer, M., *et al.* Feature-based molecular networking in the GNPS analysis environment. *Nature Methods* **2020**, *17*, 905-908. 10.1038/s41592-020-0933-6
52. Christiansen, J.V.; Larsen, T.O.; Frisvad, J.C. Production of Fungal Quinones: Problems and Prospects. *Biomolecules* **2022**, *12*, 1041. 10.3390/biom12081041
53. Ruttkies, C.; Schymanski, E.L.; Wolf, S.; Hollender, J.; Neumann, S. MetFrag relaunched: incorporating strategies beyond in silico fragmentation. *J. Cheminf.* **2016**, *8*, 3/1. 10.1186/s13321-016-0115-9
